# Characterization of a synthetic infectious cloned DNA of simian foamy virus serotype 1

**DOI:** 10.1038/s44298-025-00162-5

**Published:** 2025-12-01

**Authors:** Sandra M. Fuentes, Nicholas Mattson, Trent J. Bosma, Eunhae H. Bae, Arifa S. Khan

**Affiliations:** 1 Laboratory of Retroviruses, Division of Viral Products, Office of Vaccines Research and Review, Center for Biologics Evaluation and Research, U.S. Food and Drug Administration, Silver Spring, MD USA; 2 Present Address: University of Delaware, Newark, DE USA; 3 Present Address: University of Arkansas for Medical Sciences, Northwest Regional Campus, Internal Medicine, Little Rock, AR USA

**Keywords:** Genetics, Microbiology, Molecular biology

## Abstract

A virus stock of a cloned infectious DNA of simian foamy virus serotype 1 (SFV-1) was obtained in *M. dunni* cells (designated as pSFV-MD). The kinetics of replication were similar to the parent uncloned SFV-MD stock in *M. dunni* cells and in FRhK-4 cells, but in Vero cells, pSFV-MD showed a similar level of reverse transcriptase activity, but a faster progression of CPE. HTS variant analysis of pSFV-MD and SFV-MD passaged through Vero cells showed accumulation of G-to-A mutations in the *bel2* and *tas* genes. Some of the mutations created stop codons, truncating Tas and Bet proteins. The generation of genomic variants in SFV-1 was identified by passaging the pSFV-MD genetically homogenous virus stock in Vero cells. The accumulation of APOBEC SNVs in the SFV-1 genome highlights the species-specific interactions of Bet and APOBEC proteins since the macaque SFV-1 Bet could not effectively counteract the AGM APOBEC activity in Vero cells.

## Introduction

Spumaretroviruses (commonly referred to as foamy viruses; FVs) are found in a variety of mammals including simian, bovine, feline, equine, and chiropteran^1–7^. Intra- and inter-species transmission of simian foamy viruses (SFVs) has been reported in non-human primates (NHPs) due to the prevalence of circulating viruses. Cross-species transmissions of SFVs to humans have occurred due exposure to infected NHPs, including bites from infected nonhuman primates or consumption of infected meat^8–21^. Human infections have not been seen with FVs of other animal species, even those in close contact with humans, such as bovine or feline. FV infections in vivo are generally latent, and currently there is no evidence of disease in the natural host or upon cross-species infection. In NHPs, virus expression is seen in oral tissues^22,23^, and shedding may result in the intra-species circulation of viral “swarms” and inter-species generation of recombinant viruses in NHPs^20,24^.

We previously identified that SFV serotype 2 (SFV-2 or SFVmcy_FV34) was a recombinant virus involving SFV serotype 1 (SFV-1 or SFVmcy_FV21)-like sequences from a macaque and *env* RBD sequences from SFV of an African green monkey^25^. This discovery was unexpected since both SFV-1 and SFV-2 were isolated from kidney tissues of the same monkey species (*Macaca cyclopsis*) and seemed to have similar biological properties^6^. The results suggested that recombination may be occurring more frequently in FVs and perhaps contributing to increasing the viral diversity of circulating viruses and expanding virus transmission. The majority of SFV research has focused on the prototype FV isolate (PFV), which was isolated from a human but was found to be of chimpanzee origin (currently designated as SFVpsc_huHSRV.13)^26,27^.

Reagents for extensive SFV research are still limited or have not been developed. The first FVs were discovered in tissues of two Taiwanese monkeys (*M. cyclopsis*) and designated based on serotyping as SFV-1 and SFV-2, followed by SFV-3 (currently designated SFVcae_FV2014), which was isolated from an African green monkey^5,6^. Although the sequences and biological properties of these viruses have been well-studied, there is little understanding of the viral-host interactions resulting in differences in virus replication in cell lines of different species and tissues, and the viral-viral interactions involved in recombination of SFVs^5,6,25,28–30^.

We have used high-throughput sequencing (HTS) to obtain the consensus sequence of our uncloned SFV-1 laboratory stock, which was grown in *Mus dunni* cells (designated as SFV-MD in this study). The SFV-MD consensus sequence (SFVmcy_FV21 submitted to Genbank; NCBI accession no. MN585198) was used to generate a cloned infectious synthetic DNA (designated as pSFV in this study) and produce a genetically homogenous virus stock in *M. dunni* cells (designated as pSFV-MD). We have compared the replication kinetics of pSFV-MD and SFV-MD in cell lines of different species and performed HTS variant detection analysis to identify the generation of mutations in the FV genome and potential impact on virus replication. Our results showed cytidine deaminase activity of the APOBEC (apolipoprotein B mRNA editing catalytic polypeptide-like) protein family was the major contributor to the generation of species-specific single-nucleotide variants (SNVs).

## Results

### Development of a cloned infectious SFV-1 DNA (pSFV) and preparation of the pSFV-MD virus stock

A cloned DNA was initially synthesized in our laboratory based on the only published SFV-1 full genome sequence^29^ (accession number X54482), but this was found to be non-infectious. Therefore, we sequenced our laboratory uncloned SFV-MD virus stock by HTS and obtained a consensus sequence by mapping the reads to the published reference sequence. The SFV-1 consensus sequence obtained from the SFV-MD virus stock was submitted to Genbank (NCBI accession no. MN585198; SFVmcy_FV21). The SFV-MD consensus genome length (12,972 bp) and the position of open reading frames (ORFs) were the same as the reference sequence^29^ (Table 1).

**Table 1:** SFV-MD genome annotation

LTR/gene	Nucleotide position (nt)^a^	Encoded amino acids
5’ LTR	1..1621	-
*gag*	1737..3680	647
*pol*	3628..7077	1149
*env*	7007..9976	989
*tas*	9946..10872	308
*bel2*	10493..11704	- ^b^
3’ LTR	11352..12972	-

^a^Based on accession no. MN585198.

^b^Spliced *tas/bel2* transcript *bet* encodes 487 amino acids.

Comparison of the SFV-MD consensus sequence and the reference sequence indicated 99.72% identity; the 29 nucleotide differences were distributed in the genome and resulted in 14 missense mutations (data not shown).

**Fig. 1:**
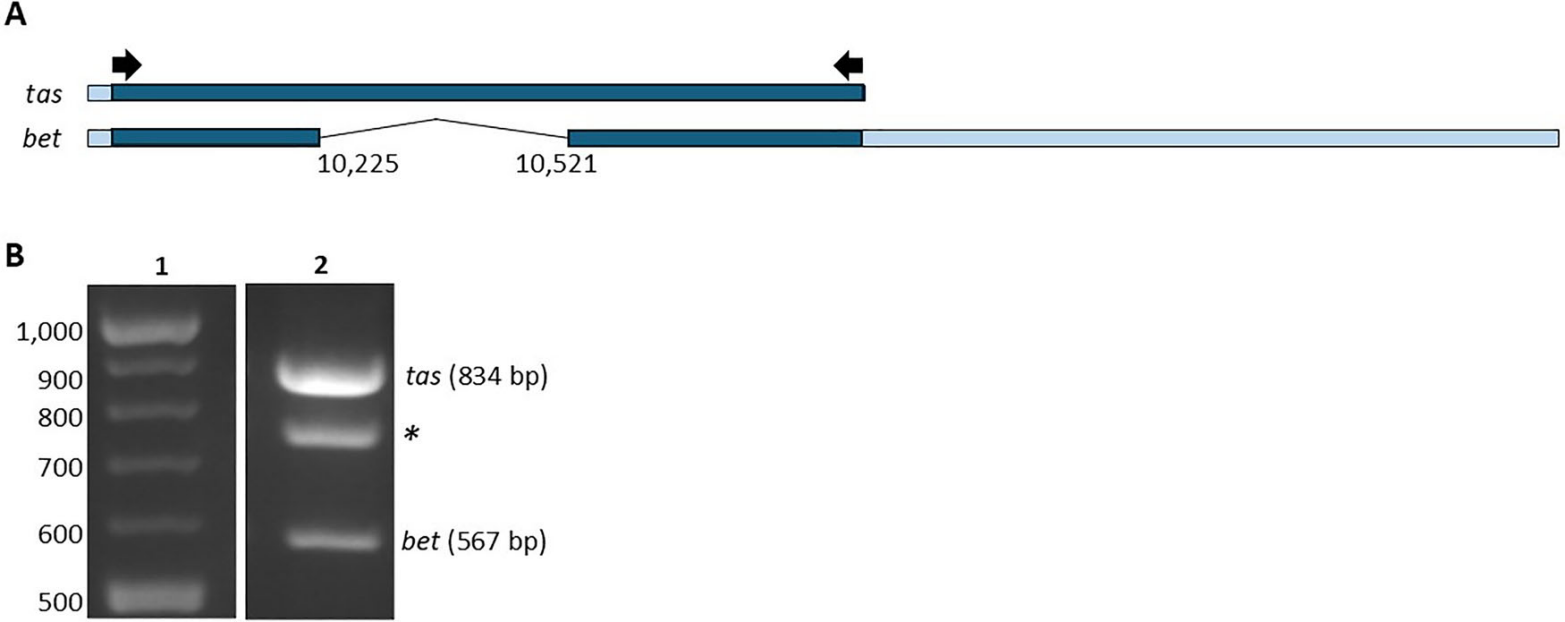
RT-PCR analysis of *tas* and *bet* transcripts. Total RNA was extracted from supernatant collected at day 9 of SFV-MD infection in *M. dunni* cells (Fig. 2A) and RT-PCR-amplified using primers located outside the splice sites (described in Materials and Methods). **A** *tas* and *tas/bel2 (bet)* transcripts are depicted, indicating the PCR-amplified regions (dark blue bar); location of the forward and reverse primers (black arrows), and the nucleotide position of the splice sites in *bet* (based on the SFV-MD consensus sequence). **B** Agarose gel analysis of the RT-PCR amplicons. Lane 2 shows the DNA corresponding to *tas* and *bet* (834 bp and 567 bp, respectively); the asterisk indicates mixed sequences of the *tas* and *bet* transcripts. Lane 1, 100-bp DNA ladder.

To identify the *tas/bel2* splice sites in the SFV-MD genome, which create the *bet* transcript, the HTS reads were aligned to the SFV-1 reference genome (accession number X54482) and reads with unaligned ends in the *tas* and *bel2* regions^31^ were identified at nucleotide positions 10225 and 10521, confirming previous reports for the location of the donor and acceptor splice sites in the *bet* transcript^32^. The donor and acceptor splice sites were verified by mapping the reads to the *bet* transcript (nt 9946..10225^10521..11704). Over 120,000 reads (from the MiSeq and NextSeq data) mapped to the spliced transcript, thus verifying the position of the *tas/bel2* splice sites. The splice site was further confirmed by RT-PCR analysis of the SFV-MD stock passaged in *M. dunni* cells using oligonucleotides targeting the sequences outside the splice junction, followed by Sanger sequencing of the RT-PCR amplified DNA fragments (Fig. 1) and blast analysis against the reference genome.

The consensus sequence of the SFV-MD was used to synthesize a cloned DNA of SFV-1 designated as pSFV in this study, which was shown to be infectious by transfection of MD cells. A virus stock was prepared in *M. dunni* cells and designated as pSFV-MD. The titer of the pSFV-MD virus stock was determined to be 10^4.83^ TCID_50_/mL. HTS confirmed that the consensus sequence of the pSFV-MD virus stock was identical to the sequences of the pSFV DNA and to the consensus SFV-MD sequence, indicating that no mutations arose during preparation of the pSFV-MD virus stock.

## Infectivity analysis of pSFV-MD and SFV-MD

**Fig. 2:**
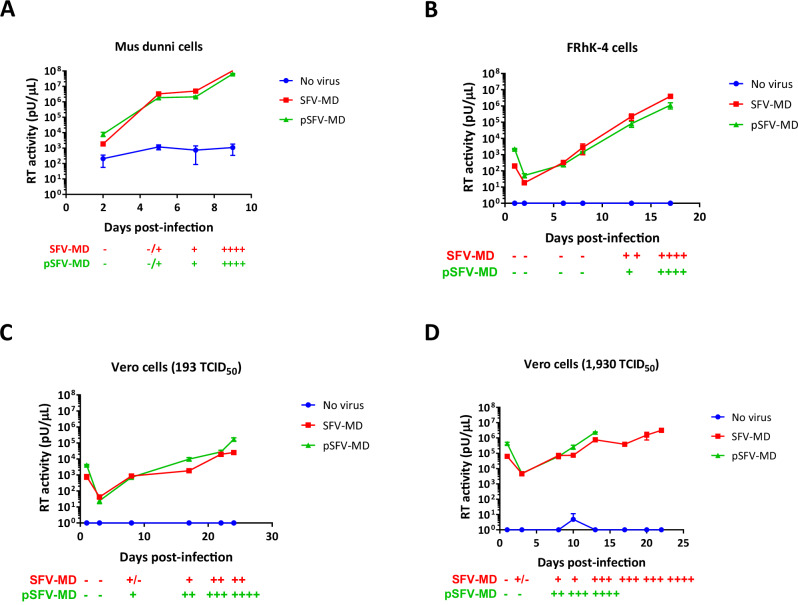
Growth kinetics of SFV-MD and pSFV-MD. Cell lines *M. dunni* (**A**), FRhK-4 (**B**) and Vero (**C**, **D**) were infected with SFV-MD (red squares) or pSFV-MD (green triangles) at 193 TCID_50_ (**A**–**C**) or 1930 TCID_50_ (**D**). The negative controls were uninfected cells (blue circles). Virus replication was measured in cell free supernatant by the PERT assay and appearance of CPE in the cell monolayer (−/+, a few syncytia; +, CPE up to 25%; ++, up to 50%; +++, up to 75%; and ++++, > 75% of cell monolayer affected). The last RT data point indicates culture termination due to CPE in the infected cells (<25% monolayer remaining due to cell death; 4+).

The replication kinetics of the pSFV-MD and SFV-MD virus stocks were compared by infection of *M. dunni* cells (mouse fibroblasts), FRhK-4 (rhesus monkey epithelial cells), and Vero (African green monkey epithelial cells). All cell lines were infected with a low infectious titer (193 TCID_50_) to avoid rapid CPE and enable the measurement of a virus growth curve. Virus replication was monitored by observing CPE in the adherent cells and measuring RT activity produced in cell-free supernatant (Fig. 2, panels A-C).

pSFV-MD had similar replication kinetics to SFV-MD in *M. dunni* and FRhK-4 cells, with a more rapid replication kinetics and higher RT level in *M. dunni* cells (terminal CPE and peak RT activity was reached at day 9 in *M. dunni* cells and at day 17 in FRhK-4 cells). Furthermore, the infectious virus titers were higher for SFV-MD and pSFV-MD passaged in *M. dunni* cells than in FRhK-4 cells (Table 2).

**Table 2:** Comparison of infectious virus titers of SFV-MD and pSFV-MD in different cell lines

Cell line	Log10 TCID_50_/mL^a^	
	SFV-MD	pSFV-MD
*Mus dunni*	5.50	4.83
FRhK-4	4.83	4.50
Vero (193 TCID_50_)	2.50	2.20
Vero (1930 TCID_50_)	2.50	2.17

^a^TCID_50_ titers were determined from the supernatant of the last day of culture for each sample in the cell line indicated.

In Vero, the pSFV-MD showed similar RT production as SFV-MD but resulted in different progression of CPE: pSFV-MD reached terminal CPE (4+) on day 24, whereas the infection with the SFV-MD only progressed to 2 + CPE, when both cultures were terminated (Fig. 2C). To confirm the difference in CPE observed between pSFV-MD and SFV-MD in Vero cells, the replication kinetics of the viruses were evaluated by infection with 10-times more virus, equivalent to 1930 TCID_50_ (Fig. 2D). For both viruses, the RT activity was higher with the high dose in Vero cells than the low dose infection, as expected, and was comparable to that seen in the FRhK-4 cells. Additionally, both viruses reached the 4+ terminal CPE, although at different times during the culture period. The culture infected with pSFV-MD had rapid CPE reaching 4+ by day 13, whereas the CPE with SFV-MD resulted in culture termination at day 22. Both the Vero cell-passaged SFV-MD and pSFV-MD had similar and low titers using either the low or high dose of inoculum (Table 2). Therefore, the higher inoculum resulted in more rapid CPE and higher RT activity without an increase in infectious virus titers, indicating the higher RT was associated with the production of more cell-associated and noninfectious virus^33^. Together, these results suggest that SFV-MD and pSFV-MD have similar kinetics of replication in permissive cells like *M. dunni* and FhRK-4 cells but not in cells less permissive to SFV-1 replication, like Vero^34^. It was noted that SFV-MD had slightly higher infectious virus titers in all the cell lines than pSFV-MD (Table 2). Since pSFV-MD was derived from the consensus sequence of SFV-MD, the difference in replication may be due to the presence of genome variants in the uncloned SFV-MD stocks that were absent in pSFV-MD.

## Variant detection analysis of SFV stocks

**Fig. 3:**
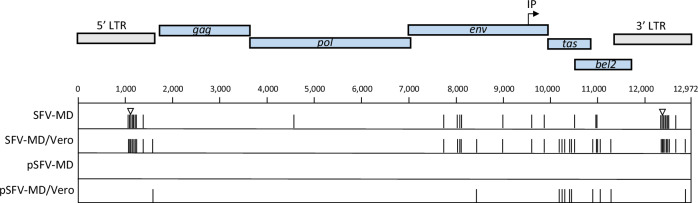
Whole genome variant detection in SFV-1 virus stocks. SNVs were identified in the SFV-MD, SFV-MD/Vero and pSFV-MD/Vero virus stocks as described in Materials and Methods and are indicated in the SFV-1 genome (black vertical line). The 8 nt deletion in the LTRs is indicated by the open triangle.

The viral sequence diversity in the SFV-MD stock was evaluated by the detection of sequence variants in the full-length genome using the HTS data that was obtained for determining the consensus SFV-MD genome sequence. The results of the short read analysis revealed 19 single-nucleotide variants (SNVs) with ≥ 20% frequency in the SFV-MD stock. These were localized mostly in the *env* and LTRs (7 and 8 SNVs, respectively), although 1-2 SNVs were found in *pol*, *bel2*, and in the overlapping *tas*/*bel2* region (SFV-MD in Fig. 3). Additionally, an 8 nt deletion was identified in the Tas responsive element (TRE) in the LTRs. This was confirmed with long read sequencing, which identified two major LTR sequence variants in SFV-MD, comprising of a complete LTR or an LTR with an 8 nt deletion. This deletion was found in 45% of the long reads covering the 5’ or 3’ LTR (in 24% of the short reads; Table 3). Deletions in the U3 region of the SFV LTRs have been shown to involve the direct repeats in tissue-culture gown viruses^35,36^, however, no direct repeats were found around the 8 nt deletion in the U3 region of the SFV-MD LTRs, suggesting a different mechanism. The G -to-A SNVs were distributed in both LTR variant populations.

**Table 3:** Sequence variants detected in SFV-1 virus stocks

Viral region	Variant Position (nt)	Variant (Reference > Allele)	Amino acid change (Protein)	Variant Frequency (%)			
				SFV-MD	pSFV-MD	SFV-MD/Vero	pSFV-MD/Vero
5’ LTR-U3	1020	C > T	-	38.81	-	25.31	-
	1042	T > G	-	41.04	-	67.2	-
	1058..1065	ΔTGTAAAAT	-	23.87	-	-	-
	1068	G > A	-	37.88	-	82.72	-
	1080	G > T	-	-	-	27.28	-
	1107	G > A	-	28.57	-	-	-
	1114	G > A	-	46.12	-	46.97	-
	1162	G > A	-	32.2	-	20.95	-
	1172	G > A	-	23.85	-	25.74	-
5’ LTR-R	1330	C > T	-	43.51	-	34.28	-
5’ LTR-U5	1533	G > A	-	-	-	30.24	26.65
*pol*	4539	G > A	-	22.78	-	-	-
*env*	7733	A > G	T243A (SU)	24.87	-	24.44	-
	8031	G > A	R342Q (SU)	20.38	-	23.26	-
	8074	G > A	-	32.98	-	35.41	-
	8102	A > G	K366E (SU)	20.29	-	34.29	-
	8426	G > A	G474R (SU)	-	-	25.41	26.85
	8991	G > A	R662K (TM)	33.64	-	28.65	-
	9610	C > T	-	23.91	-	24.74	-
	9876	T > C	F957S (TM)	26.64	-	28.5	-
*tas/bet*	10202	G > A	W86* (Tas, Bet)	-	-	32.46	32.39
	10257	G > A	-	-	-	41.67	35.78
	10316	G > A	W124* (Tas)	-	-	33.56	29.67
	10430	G > A	W162* (Tas)	-	-	26.89	28.33
	10461	G > A	-	-	-	42.84	39.46
	10525	A > G	K194E (Tas)	62.74	-	35.77	-
	10902	G > A	W221* (Bet)	-	-	40.55	33.64
	10988	G > A	V250I (Bet)	23.84	-	24.12	-
	11004	T > C	L255P (Bet)	49.37	-	59.44	-
	11063	G > A	G275R (Bet)	-	-	32.16	28.29
	11294	G > A	G352R (Bet)	-	-	41.24	34.05
3’ LTR-U3	12371	C > T	-	38.91	-	25.56	-
	12393	T > G	-	41.22	-	66.04	-
	12409..12416	ΔTGTAAAAT	-	23.78	-	-	-
	12419	G > A	-	38.15	-	81.99	-
	12431	G > T	-	-	-	26.33	-
	12458	G > A	-	28.23	-	-	-
	12465	G > A	-	46.53	-	47.27	-
	12513	G > A	-	32.54	-	20.83	-
	12523	G > A	-	23.92	-	26.32	-
3’ LTR-R	12681	C > T	-	43.3	-	33.29	-
3’ LTR-U5	12884	G > A	-	-	-	28.2	24.86

-, no amino acid change or variant frequency is ≤ 20%.

*, stop codon.

Δ, deletion.

No SNVs were detected in the pSFV-MD, even when the threshold for minimum variant frequency was lowered from 20% to 1%. This indicated that 3 passages of the virus in *M. dunni* cells did not create genome variants and confirmed that the pSFV-MD was a genetically homogenous virus stock.

To assess whether sequence variants contributed to the difference in the replication kinetics of pSFV-MD and SFV-MD in Vero cells, virus supernatant was collected from the infectivity experiments at culture termination (Fig. 2D), and the variants detected by HTS were compared to those in the inocula. Twenty-eight variants were identified in the virus collected from the SFV-MD/Vero infection (Fig. 3), which included 11 that were newly generated by passage in the Vero cells, of which 10 were present in both SFV-MD/Vero and pSFV-MD/Vero at culture termination.

**Fig. 4:**
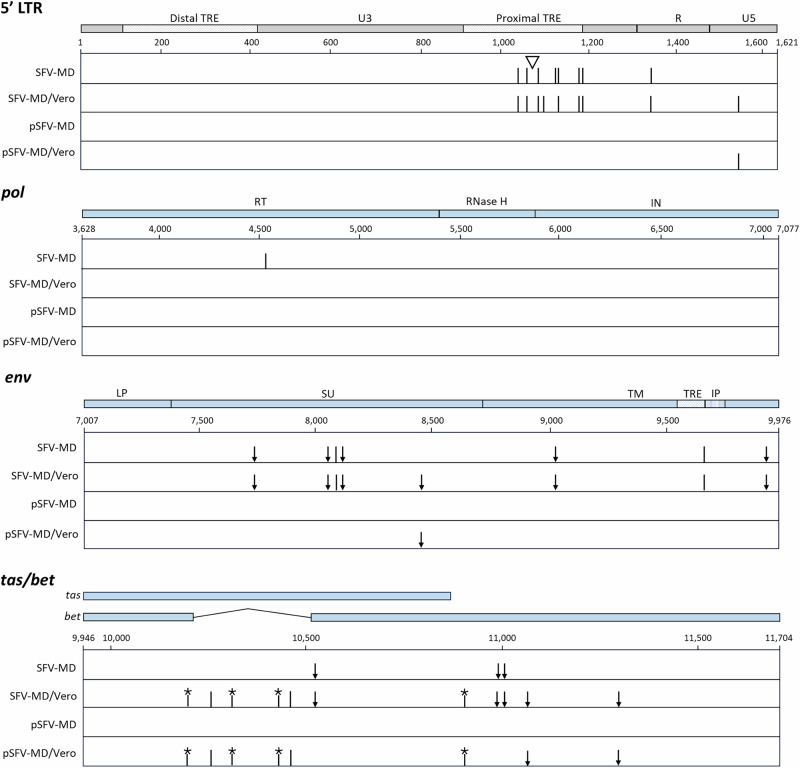
Sequence variants detected in the SFV LTR and genes. The Tas-response element (TRE) in the U3 region of the 5’ LTR is indicated, and the 8 nt deletion is shown by the open triangle. The proteins encoded by the genes are indicated. The location of the TRE and internal promoter (IP) are in *env* are indicated. SNVs in the 5’LTR, *pol*, *env*, and *tas/bet* were identified by HTS variant detection analysis of the SFV-MD, SFV-MD/Vero, pSFV-MD, and pSFV-MD/Vero virus stocks and are indicated for each: silent mutations, black vertical line; missense mutations, black arrow; stop mutations, asterisk.

The results of the variant detection analysis in the 5’ LTR and individual viral genes are shown in Fig. 4. The changes seen in the 5’LTR were also present in the 3’ LTR. The SNVs in the LTR in the SFV-MD and in the SFV-MD/Vero were mostly within the predicted promoter proximal TRE (nt 893..1171), which is important for transactivation and replication of FVs^37^. It was noted that the 8 nt deletion was not seen in the LTRs of SFV-MD/Vero and pSFV-MD/Vero (<1%), and a new mutation was detected in the U5 region of the LTR. No sequence variants were found in *gag*; and only one mutation was detected in the RT in the *pol* of SFV-MD. In *env* all the variants were detected in the transmembrane (TM) and surface (SU) proteins. Importantly, most of the new mutations in pSFV-MD/Vero and SFV-MD/Vero were introduced in the Tas and Bet proteins, which are critical for regulating virus replication.

The mutations identified by short-read sequencing and their positions in the viral regions are shown in Table 3. Most of the variants in SFV-MD and SFV-MD/Vero were present at a similar frequency; however, there were some differences in the two stocks. An 8 nt deletion in LTR was present at 24% frequency in the SFV-MD stock and <1% frequency in the SFV-MD/Vero stock. Additionally, mutations at nt position 1107/12458 in the LTRs and at 4539 in *pol* were found in the SFV-MD stock but not in the SFV-MD/Vero stock. Since there was no selection for these mutations upon passage in Vero cells, these were not likely contributing to the difference in the SFV replication kinetics in Vero and *M. dunni* cells (Fig. 2). In contrast, the variants at nt positions 1042/12393 and 1068/12419 in the LTRs, and 11004 in Bet changed from a minority population in SFV-MD to become the main sequence variant present in the SFV-MD/Vero stock, suggesting the selection of these variant sequences during virus replication in Vero cells.

Interestingly, most of the variants in the SFV virus stocks were G-to-A, suggesting they were generated by the APOBEC family of cytidine deaminases. All of the new sequence variants generated in SFV-MD/Vero (except one) and in the pSFV-MD/Vero were G-to-A: one in the *env* region resulted in an amino acid mutation (G474R) in the SU protein. Most of the new variants found in the SFV-MD/Vero virus stock were found in the region encoding for the Tas and Bet proteins. Three of these were missense mutations, and 4 of them resulted in stop codons affecting either Tas, Bet, or both proteins. Since the Tas protein is necessary for virus expression by transactivation in the LTR, termination mutations in Tas will negatively affect virus replication. These results suggest that the low virus replication of SFV-1 in Vero cells may be due to hypermutation by APOBEC. Furthermore, the slower kinetics of replication of the SFV-MD compared to the pSFV-MD in Vero (based on CPE) suggest that viral diversity may be affecting infection/replication of the SFV-MD stock.

## Discussion

The available virus stocks of SFVs have been generated by laboratory passage of the original monkey isolates in cell lines of different species^5,6^. While the foamy virus genome is fairly stable^9,38^, it is known to be susceptible to hypermutations by APOBEC3 proteins^39,40^, in the absence of specific binding with the FV Bet protein, which may be species-dependent. The SFV-1 used in our study had an extensive passage history in several heterologous cell lines, which include human, monkey, rabbit, rat, and dog, prior to the amplification in *M. dunni* cells to generate our laboratory SFV-MD virus stock. Therefore, SFV-MD is expected to contain viral variants, but it is unknown when mutations might have been acquired during the passage history of the virus. Using such uncloned virus stocks in biological studies can lead to confounding results regarding virus-host interactions and virus fitness. Although a few infectious SFV DNAs have been made^32,41,42^, including PFV^43^, studies of the virus biology, such as cell tropism, host range, and replication have used uncharacterized virus stocks. In this study, an infectious pSFV DNA was synthesized based on the HTS consensus sequence of our laboratory stock of SFV-MD and generated a virus stock (pSFV-MD) by transfection and three passages in *M. dunni* cells. Characterization of the pSFV-MD virus stock indicated a lower titer than the “parent” SFV-MD virus stock (10^4.83^ and 10^5.5^, respectively), although both stocks had similar RT activity (1.6 × 10^8^ pU/mL and 1.2 × 10^8^ pU/mL, respectively). It should be noted that in our study, the results comparing the replication kinetics and generation of mutations in the cloned pSFV-MD and the uncloned SFV-MD were obtained by inoculating cells based on the infectious titer.

Sequence variant analysis of the HTS reads indicated that the pSFV-MD virus did not acquire any SNVs during the generation of the stock by three passages in *M. dunni* cells whereas the SFV-MD stock had many SNVs, located mostly in the LTR and *env*, with a few in the *tas* and *bel2*, which could be due to the passage history of the SFV-1. Sequence analysis indicated that most of the mutations were G-to-A changes, which were likely generated due to APOBEC hypermutation in the virus genome^44^. The role of human APOBEC in the hypermutation of HIV-1 has been well studied. In this case, the Vif protein has been shown to inhibit this hypermutation by targeting the APOBEC3 proteins for degradation^45–51^. More recently, human APOBEC3 proteins have also been shown to restrict replication of the feline foamy virus and PFV^52–57^. Our results corroborate the previous results of APOBEC hypermutation in FVs and extends it to detection in the SFV-1 rhesus macaque isolate. Although less is known about APOBEC hypermutation of the FV genome compared to HIV-1, the FV Gag protein has been shown to bind to human APOBEC A3G and A3F and aid in the incorporation of these proteins into the virions, while the FV Bet protein acts as an APOBEC antagonist, similar to the HIV-1 Vif, and prevents APOBEC protein incorporation into the virions^52,55,56^.

To investigate the effects of hypermutation by APOBEC3 of different species on SFV, we evaluated virus replication of the genetically homogenous pSFV-MD virus stock in mouse and monkey cell lines. We compared the kinetics of replication of the pSFV-MD virus stock and uncloned SFV-MD virus stock, which contained a swarm of variant genomes, in infectivity studies using cell lines of different species: *M. dunni*, FRhK-4, and Vero cells. The results in *M. dunni* and FRhK-4 cells did not indicate significant differences in the RT activity, CPE, or infectious virus titer between the two virus stocks. In Vero cells, although both virus stocks reached similar levels of cell-free RT activity, the pSFV-MD virus showed more rapid progression of CPE than SFV-MD. The difference in the kinetics for the development of CPE observed between SFV-MD and pSFV-MD may be due to the large number of mutations identified in the LTR and *env* in the SFV-MD stock, which may negatively impact virus replication in Vero cells. Of note, most of the mutations (4/6) in *env* were in the receptor binding domain (RBD) and the arginine residue at position 342 (Table 3), corresponding to lysine at position 342 in the PFV, has been shown to be important for binding of PFV to the cellular receptor, heparan sulfate^58^.

Molecular and phylogenetic studies show ancient co-evolution of SFVs with their nonhuman primate species^59^. Generally, genetically distinct SFV strains co-circulate with their species, however, inter-species transmission and cross-species infection of humans can occur by exposure to infected animals or tissues^60,61^. The factors regulating the generation of virus diversity and virus transmission are complex and have not been well-studied. In this study, we analyzed the generation of variants in the SFV-1 macaque isolate by passage in cell lines from mouse, rhesus macaque, and African green monkey. HTS variant analysis on the viruses obtained after passage in Vero cells (pSFV-MD/Vero and SFV-MD/Vero) showed that both the pSFV-MD and the SFV-MD virus stocks acquired similar G-to-A hypermutations, indicating that the rhesus SFV-1 Bet could not counteract the African green monkey APOBEC activity. Most of the mutations were in the regulatory *tas* and *bel2* genes and resulted in truncated and likely non-functional proteins and defective viruses. These results suggest that APOBEC may have a potential role in inter-species transmission of SFV in different monkey species (e.g., rhesus vs African green monkeys) and possibly cross-species transmission to humans. The results of this study highlight the use of a cloned, well-characterized virus stock for advancing studies of virus biology and investigations of virus-host interactions involved in regulating virus replication and transmission.

## Methods

### Preparation and characterization of the uncloned virus stock of SFV-MD

SFV-1 was obtained from the American Type Culture Collection (ATCC, catalog number VR-276, lot 215340, reference lot 5 WE). The passage history of the ATCC virus, which was isolated from *Macaca cyclopis* #21 (designated as FV-21) in the U.S. Medical Research Unit #2, Taiwan^6^, was provided as follows: initially, 13 passages in primary rabbit kidney (PrRabK) cells, 1 passage in the human KB cell line (currently known to be a subline of HeLa), 7 passages in the rhesus monkey kidney LLC-MK2 cell line, 1 passage in KB (spinner), 2 passages in the rat kidney RatK cell line, 3 passages in the human HEp-2 cell line (currently known to be derived from HeLa contamination) and finally 2 passages in the dog A-72 cell line. SFV-1 has been designated as SFVmcy_FV21 based on the taxonomy and nomenclature^62^.

The SFV-MD virus stock was prepared in our laboratory by low passage (3 passages) of SFV-1/ATCC in *M. dunni* cells^33,63^. Cells were grown in complete medium consisting of Dulbecco’s modified Eagle medium (DMEM; Gibco, catalog no. 11995-065) supplemented with 10% heat-inactivated Fetal bovine serum (FBS; HyClone, catalog no. SH30071.03), 100 U of penicillin per mL and 100 µg of Streptomycin per mL (Quality Biological, Inc., Gaithersburg, MD; catalog no. 120-095-721), and 2 mM L-glutamine (Quality Biological, Inc., catalog no. 118-084-721). Supernatant was collected and filtered (0.45 µm) when extensive cytopathic effect (CPE) was observed, which corresponded with peak reverse transcriptase (RT) activity. The 50% tissue culture infectious dose (TCID_50_) was determined to be 10^5.5^ per mL by infection of MRC-5 cells with a 10-fold serial dilution of the virus stock and calculation of TCID_50_ using the Kärber method^64^ with a CPE readout at 13 days post-infection^65^.

For HTS sequencing, two milliliters of the virus stock were concentrated to 140 μL using a Nanosep centrifugal device with 30 K Omega membrane (Pall Corporation, Cytiva Life Sciences; Marlborough, MA). Nucleic acid extraction was done using the QIAamp Viral RNA Mini Kit (QIAGEN; catalog no. 52904) without carrier RNA. Extracted nucleic acids were sent to CD Genomics (Shirley, NY) for library preparation and HTS sequencing at 2 × 250 paired-end read format using the Illumina MiSeq platform (San Diego, CA). Adapter-trimmed reads (4,111,478) were obtained from CD genomics. The QIAGEN CLC Genomics Workbench (v12.0) was used to map the reads against the previously published SFV-1 complete genome as a reference sequence^29^ (accession number X54482.1) using default settings (at 0.8 length fraction and 0.9 similarity fraction). The LTRs were mapped separately using the same parameters. A consensus sequence of the full-length SFV-MD virus genome was obtained from the mapping of 2,012 reads with an average coverage depth of 38 reads per base. This SFV-1 genome sequence was deposited to NCBI under accession number MN585198 (SFVmcy_FV-21).

The open reading frames (ORFs) were identified using the ORF finder tool (https://www.ncbi.nlm.nih.gov/orffinder/). The splice sites in *tas/bel2* (encoding Bet) were located based on mapping of the HTS reads to the SFV-1 reference genome, which identified reads with unaligned ends at the donor and acceptor splice sites. The splice site was confirmed by an RT-PCR assay. RNA (10 µl) was used for cDNA synthesis by the iScript cDNA synthesis kit from Bio Rad Laboratories (Hercules, CA; catalog no. 1708890) following the manufacturer’s protocol. The PCR assay was performed in a 50 µL reaction containing 1X Ex Taq buffer with 0.8 mM dNTPs (Takara Bio USA, San Jose, CA; catalog no. RR001A), 5U of the Takara Ex Taq DNA polymerase, 1:100 dilution of the cDNA, and 1uM each of the SFV-1-F-9986 (forward) and SFV-1-R-10819 (reverse) primers (5’-TACATCATCTACCAGAG-3’ and 5’-AAATTGAGTCACCATAC-3’, respectively). The PCR conditions were: 94 °C for 2 min and 35 cycles of 98 °C for 10 s, 55 °C for 30 s and 72 °C for 1 min, followed by 72 °C for 10 min. The PCR product was separated in 2% low-melting-point agarose gel stained in 1X Tris-acetate-EDTA buffer (TAE, Quality Biological; catalog no. 351-009-131) containing 0.5 mg/mL of ethidium bromide (Invitrogen, Waltham, Massachusetts; catalog no. 15585011). The resulting bands were excised and purified using the Zymo Research Gel DNA recovery kit (Irvine, CA; catalog no. D4007). The isolated DNA fragments were sequenced by the Sanger sequencing method^66^, and the sequence was aligned to the HTS consensus sequence of the SFV-MD (NCBI accession number MN585198; SFVmcy_FV21) by using the basic local alignment search tool (BLAST)^67^ to identify the position of the splice sites.

To evaluate the presence of viral genome variants in the SFV-MD, the virus stock was sequenced using the Illumina NextSeq technology. For this, nucleic acids were extracted from 560 µL of the virus stock using the Qiagen Viral Amp kit without carrier RNA. Library preparation and sequencing was performed by the CBER Core Facility (Silver Spring, MD) using 5 µL of nucleic acid and the Illumina TruSeq Stranded mRNA kit. The library size was determined using the Agilent Bioanalyzer DNA 1000 kit (Santa Clara, CA). A MiSeq run using 5pM of the library and the MiSeq Reagent Nano Kit v2 (300-cycles, Cat no. MS-103-1001) was performed to optimize NextSeq loading conditions. Libraries (1.1-1.3 pM) were loaded onto the NextSeq Mid-output flow cell without multiplexing. Paired-end sequencing was carried for 100 × 2 cycles using the NextSeq 500/550 sequencer. Bcl files were exported and fastq files were generated for downstream analysis. Raw reads were trimmed to remove adapters and low-quality base pairs using the CLC genomics workbench (v22.0.2). Reads with Phred score < 30 or < 50 bp in length were discarded. QC pair-ended reads were mapped to the MN585198 sequence. The QC and mapping statistics are shown in Table 4. The single nucleotide variants (SNVs) and indels in the stock were identified using the Fixed Ploidy Variant Detection Tool (CLC) with the following non-default settings: ignore positions with coverage above 500,000, do not ignore broken pairs or non-specific matches and ≥ 20% frequency. A known variants track was created by combining the variants found in all the samples. This sequence was used to determine the frequency of the known variants in all the samples using the Identify Known Mutations from Mappings Tool (CLC) using default settings.

**Table 4:** Quality check and read mapping results of HTS for the SFV-MD and pSFV-MD virus stocks

HTS Results	SFV-MD	pSFV-MD
**Illumina NextSeq**		
Raw reads	302,773,390	242,089,702
Paired QC reads	160,776,446	152,092,996
No. reads that mapped to MN585198	41,907,679	6,539,775
Length of reference genome covered (%)	12,972 bp (100%)	12,972 bp (100%)
Average coverage per base/minimum	286,772 / 77	45,990 / 17
**Nanopore Oxford Technologies MinION**		
Raw reads	3,043,020	ND^a^
No. reads that mapped to MN585198	117, 277	ND

^a^*ND* not done.

To perform long read sequencing by Oxford Nanopore Technologies, RNA was extracted from 1 mL of the SFV-MD stock using Trizol LS (Life Technologies; catalog no. 10296010) and following the manufacturer’s protocol. Double-stranded cDNA was generated using the Maxima H minus double-stranded cDNA synthesis kit (Thermo Scientific; catalog no. K2562) using random hexamers following the manufacturer’s protocol. The dscDNA was purified using the GeneJET PCR purification kit (Thermo Fisher Scientific; catalog no. K0701) and used for library preparation and sequencing (Ligation Sequencing Kit; Oxford Nanopore Technologies, Oxford, UK; catalog no. SQK-LSK114) with the following modifications: 45 µL of AMPure beads (Beckman) were used for the second DNA clean-up step together with long fragment buffer (LFB) for washing. Sequencing was performed on R10.4.1 flow cells (Catalog no. FLO-MIN114) on a MinION Mk1B for 30 h using the MinKNOW software. Sequences were base-called after sequencing had completed with DORADO version 0.9.1 using the super accurate algorithm (dna_r10.4.1_e8.2_400bps_sup@v5.0.0) with quality trimming set at Q7. Reads less than 50 base pairs were removed using Cutadapt^68^ resulting in 3,043,020 reads (Table 4). Reads were mapped to the SFV-MD consensus sequence (NCBI accession number MN585198; SFVmcy_FV21) and variants were detected using the CLC genomics workbench (v25.0.1) with the “Map Long Reads to Reference” tool using default settings. The total number of reads that mapped to the reference was 117,277. The N50 read length for the mapped reads was 1548, the mean read length was 1194, and the mean read quality was 20.27. SNVs, MNVs, and deletions were detected using the CLC genomics workbench “Fixed Ploidy Variant” detection tool.

### Preparation and HTS analysis of pSFV-MD virus stock

Initially, SFV genome DNA was synthesized (Genscript, Piscataway, NJ) using the only available full-length genome sequence of SFV-1 (NCBI accession no. X54482.1.1). Due to the lack of infectivity of this DNA, a second viral DNA was synthesized using the consensus sequence of SFV-MD obtained by HTS, cloned into pUC57-Brick (Genscript), and designated as pSFV DNA.

A virus stock was prepared by transfection of *M. dunni* cells with pSFV DNA. Cells were seeded in a 6-well plate. One well was transfected with 4 µg of pSFV mixed with 1 µg of polyethylenimine (PEI, Polysciences; Warrington, PA) and 300 µL of 150 mM NaCl in a total volume of 2 mL DMEM supplemented with 5% FBS. Eighteen-hours post-transfection, the cells were transferred to a 75 cm^2^ flask in complete media (containing 10% FBS) and passaged twice until the CPE had spread to >75% of the monolayer on day 8, when the supernatant was collected, filtered through a 0.45 µm filter, and stored in aliquots at −80 °C. The titer of the pSFV-MD was determined as 10^4.83^ TCID_50_ per mL (MRC-5, 15 days) as described above for SFV-MD. The nucleic acid extraction, library prep, sequencing and bioinformatics analysis for the pSFV-MD stock were done as described above for the Illumina NextSeq sequencing of the SFV-MD stock.

### Infectivity studies of pSFV-MD and SFV-MD stocks

Infectivity studies were done in *M. dunni*, Vero, and FRhK-4 cells. *M. dunni* and FRhK-4 cells were grown in DMEM media supplemented as described above. Vero cells were grown in Minimum Essential Media (MEM; Gibco, catalog no. 11095-080) supplemented with 10% heat-inactivated FBS, 100 U of penicillin per mL and 100 µg of Streptomycin per mL, 2 mM L-glutamine, 1 mM sodium pyruvate (Quality Biological, Inc.; catalog no. 116-079-721) and 1X MEM non-essential amino acids (MEM-NEAA; Quality Biological, Inc.; catalog no. 116-078-721). Cells were seeded in 25 cm^2^ flasks overnight and infected with SFV-MD or pSFV-MD using 193 TCID_50_ (*M. dunni*, FRhK-4, and Vero cells) or 1930 TCID_50_ (Vero cells only). Cells were transferred upon reaching confluence to 75 cm^2^ flasks and passaged every 2-3 days. At every passage the presence of CPE was visually evaluated, and filtered supernatant was collected and stored at −80 °C for determining the RT activity using a PERT assay^34,69^. The virus titers from the supernatant collected from the terminal time-point of the cell culture were determined in MRC-5 cells with a CPE readout at 13 days (described above).

For virus variant analysis, samples were collected from SFV-MD/Vero and pSFV-MD/Vero cultures at terminal time points. Extracted nucleic acids were sequenced by Illumina NextSeq technology, and the reads were analyzed for the detection of variants as described above.

## Data Availability

The cloned pSFV-MD and data that support the findings of this study are available on request from the corresponding author (A.S.K.).
